# ^18^F-FDG PET imaging for identifying the dynamics of intestinal disease caused by SFTSV infection in a mouse model

**DOI:** 10.18632/oncotarget.6645

**Published:** 2015-12-17

**Authors:** Daisuke Hayasaka, Kodai Nishi, Takeshi Fuchigami, Kazuya Shiogama, Takanori Onouchi, Satoshi Shimada, Yutaka Tsutsumi, Kouichi Morita

**Affiliations:** ^1^ Department of Virology, Institute of Tropical Medicine, Nagasaki University, Sakamoto, Nagasaki, Japan; ^2^ Leading graduate school program, Nagasaki University, Sakamoto, Nagasaki, Japan; ^3^ Department of Radioisotope Medicine, Atomic Bomb Diseases Institute, Nagasaki University, Sakamoto, Nagasaki, Japan; ^4^ Department of Hygienic Chemistry, Graduate School of Biomedical Sciences, Nagasaki University, Bunkyo-machi, Nagasaki, Japan; ^5^ Department of Pathology, Fujita Health University School of Medicine, Dengakugakubo, Kutsukake-cho, Toyoake, Aichi, Japan

**Keywords:** SFTSV, ^18^F-FDG PET imaging, mouse model, intestinal disorder, antiserum therapy, Immunology and Microbiology Section, Immune response, Immunity

## Abstract

Severe fever with thrombocytopenia syndrome (SFTS) is an emerging disease that causes fever, enteritis, thrombocytopenia, and leucopenia and can be fatal in up to 30% of cases. However, the mechanism of severe disease is not fully understood. Molecular imaging approaches, such as positron-emission tomography (PET), are functional *in vivo* imaging techniques that provide real-time dynamics of disease progression, assessments of pharmacokinetics, and diagnoses for disease progression. Molecular imaging also potentially provides useful approaches to explore the pathogenesis of viral infections. Thus, the purpose of this study was to image the pathological features of SFTSV infection *in vivo* by PET imaging. In a mouse model, we showed that ^18^F-FDG accumulations clearly identified the intestinal tract site as a pathological site. We also demonstrated that ^18^F-FDG PET imaging can assess disease progression and response to antiserum therapy within the same individual. This is the first report demonstrating a molecular imaging strategy for SFTSV infection. Our results provide potentially useful information for preclinical studies such as the elucidation of the mechanism of SFTSV infection *in vivo* and the assessment of drugs for SFTS treatment.

## INTRODUCTION

Severe fever with thrombocytopenia syndrome (SFTS) is an emerging disease that was first identified in China and later in South Korea and Japan [1-4]. The causative agent, SFTS virus (SFTSV), belongs to *the genus Phlebovirus* of *the family Bunyaviridae* and is categorized as a BSL-3 pathogen. It has been suggested that SFTSV is transmitted by Ixoded ticks, and humans and animals are infected by tick bites [5, 6]. The clinical manifestations of SFTS include fever, enteritis, thrombocytopenia and leucopenia. The disease is fatal in up to 30% of cases [1-3, 7]. However, the mechanism of disease progression is not fully understood, and there are currently no specific treatments or vaccines available. Thus, elucidating the mechanism of severe disease progression leading to death is critical to developing efficient vaccines and drugs for SFTS.

*In vivo* imaging is a powerful tool that provides dynamic information on metabolic disorders, disease progression, and drug intervention. Molecular imaging technologies, including positron-emission tomography (PET) and single photon emission computed tomography, are functional *in vivo* imaging techniques that can be combined with structural imaging techniques, such as computed tomography (CT). In particular, 2-deoxy-2-[^18^F] fluoro-D-glucose (^18^F-FDG) can be used to assess glucose metabolism and ^18^F-FDG PET/CT is currently utilized for imaging tumor, infection and inflammation in both basic studies and clinical applications [8-10].

Molecular imaging has been developed and applied in research for neurology, oncology, cardiovascular physiology, and immunology. However, molecular imaging for infectious diseases caused by highly pathogenic viruses, including biosafety level (BSL)-3 agents, has not been fully utilized because of the need for a high-level biocontainment facility [11]. It is suggested that molecular imaging potentially provides useful approaches to explore the mechanism of disease progression, to assess pharmacokinetics, and to diagnose disease progression of infectious diseases, including viral infections [9, 11]. Thus, we postulated that molecular imaging provides a powerful tool for *in vivo* approach to examine the disease progression of SFTSV infection.

Type-I interferon receptor knock-out (A129) mice provide a useful model for investigating the pathogenic mechanism of SFTSV infection *in vivo* [12, 13]. We previously showed that lethal SFTSV infection in mice led to acute clinical signs, including piloerection, slowed movement, anorexia, and severe weight loss by 2 days post-infection (pi), and all mice died by 7 days pi [13]. However, the primary cause of lethal pathology was not characterized. We further showed that post-exposure treatment with anti-serum significantly protected the animals from death [13]. Thus, we expected that the mouse model provides a useful platform to study the *in vivo* imaging of disease progression and antiviral intervention caused by SFTSV infection.

The purpose of this study was to image the pathological features of SFTSV infection *in vivo* by PET. In a mouse model, we first examined the pathological features of lethal infection with SFTSV. We next assessed whether ^18^F-FDG PET/CT imaging provides an effective approach for monitoring disease progression of SFTSV infection. We further evaluated whether the therapeutic efficacy of anti-serum treatment could be observed by ^18^F-FDG PET/CT imaging in the same individual.

## RESULTS

**Figure 1:**
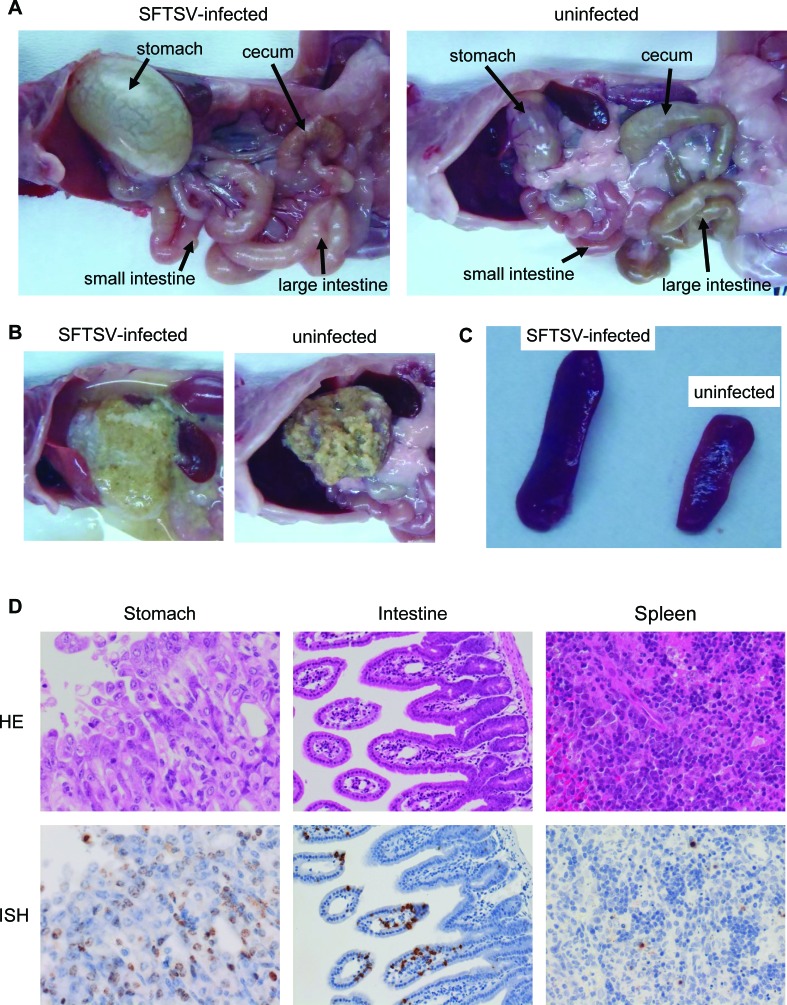
Histological features of A129 mice inoculated with a lethal dose of SFTSV **A.**-**C.** Gross pathology of gastrointestinal tracts **A.**, stomach affecting the consistency of food contents. **B.** and spleens **C.**, **D.** Histological and ISH features of the gastrointestinal tract of heavily infected mice (top panels: hematoxylin and eosin staining; bottom panels: ISH using AT-tailed antisense cocktail probes).

A129 mice were infected with a lethal dose of SFTSV, and we observed the pathological changes at 3 days pi before mice started to die at 4 days pi [13]. The gross pathology revealed that SFTSV-infected mice exhibited gastric and intestinal distensions and had reduced stools in the intestine and a reduced cecum size compared to mock-infected mice (Figure 1A). The stomach contents of SFTSV-infected mice were liquid, whereas the contents of mock-infected mice were solid (Figure 1B). A characteristic splenomegaly was present in SFTSV-infected mice compared to mock-infected mice (Figure 1C).

The histopathological examination revealed erosion in the gastric mucosa but not in the small intestinal mucosa of SFTSV-infected mice (Figure 1D). Viral genomic RNA was detected in the epithelial cells of the stomach and intestine of SFTSV-infected mice (Figure 1D). Significant histopathological changes were not observed in the spleen of SFTSV-infected mice (Figure 1D). There was no viral RNA detected in the mock-infected mice (data not shown).

These observations suggest that lethal infection with SFTSV caused severe gastrointestinal disorders. These findings suggest that the gastrointestinal tract is a potential target for PET imaging of SFTSV infection.

**Figure 2:**
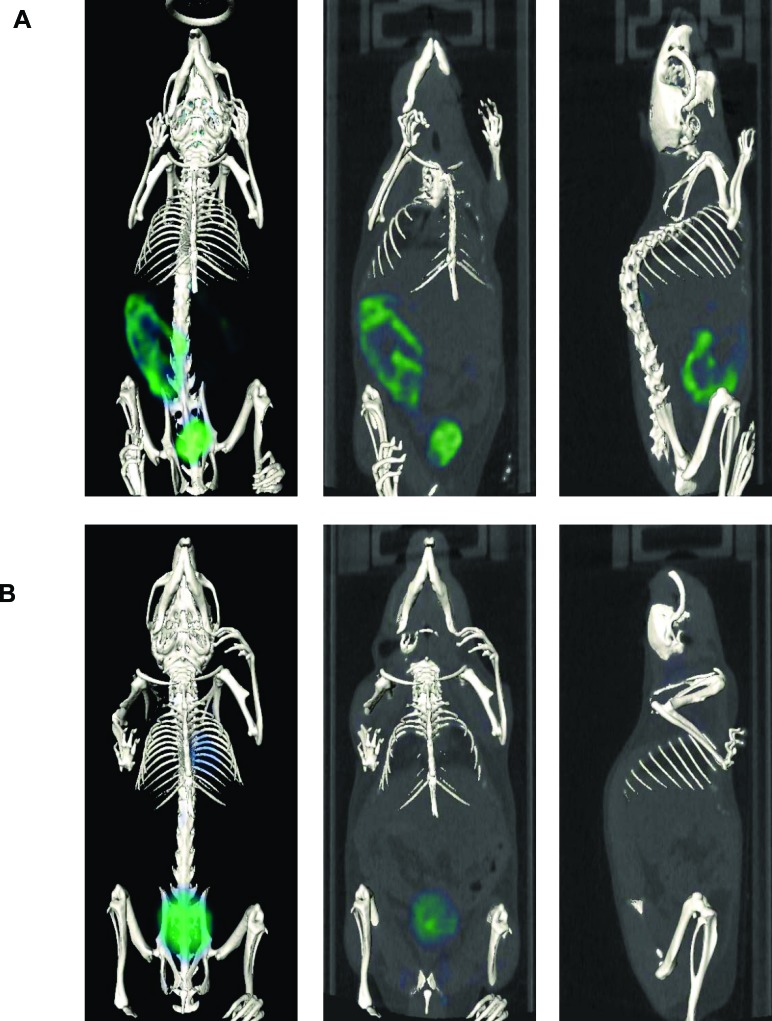
Representative whole body PET/CT images of SFTSV-infected **A.** and mock-infected A129 mice **B.** at 3 days pi. PET/CT images were acquired 30-60 min after intravenous injection of ^18^F-FDG (10 MBq in 400 μl of saline per mouse).

We next performed ^18^F-FDG PET imaging of SFTSV-infected mice at 3 days pi. ^18^F-FDG accumulation was clearly observed in the intestine of SFTSV-infected mice, but not in the stomach (Figure 2A and Video 1). The mock-infected mice did not exhibit any ^18^F-FDG accumulation in the stomach or intestine (Figure 2B and Video 2). There was no specific accumulation of ^18^F-FDG in the spleen of SFTSV-infected mice that exhibited splenomegaly (Figure 2A and Video 1). There was ^18^F-FDG accumulation in the bladders of both SFTSV-infected and mock-infected mice, indicating that the accumulation in these organs is a physiological property of ^18^F-FDGz.

These observations strongly indicate that ^18^F-FDG accumulation reflected the pathological region in the intestinal tract and could represent the characteristic features of intestinal disease following SFTSV infection in mice.

The major advantages of molecular imaging include its noninvasive nature and its ability to be used for real-time monitoring *in vivo* within the same individual. We previously showed that post-exposure treatment with antiserum at 1 hour to 3 days pi could rescue mice from lethal infection with SFTSV [13]. Therefore, we performed ^18^F-FDG PET imaging in SFTSV-infected mice treated with antiserum to identify the dynamics of intestinal disease and evaluate the therapeutic effects. To observe the sequential progress of ^18^F-FDG accumulation in mice, PET imaging were performed at 2 and 4 days pi in each mouse.

**Figure 3:**
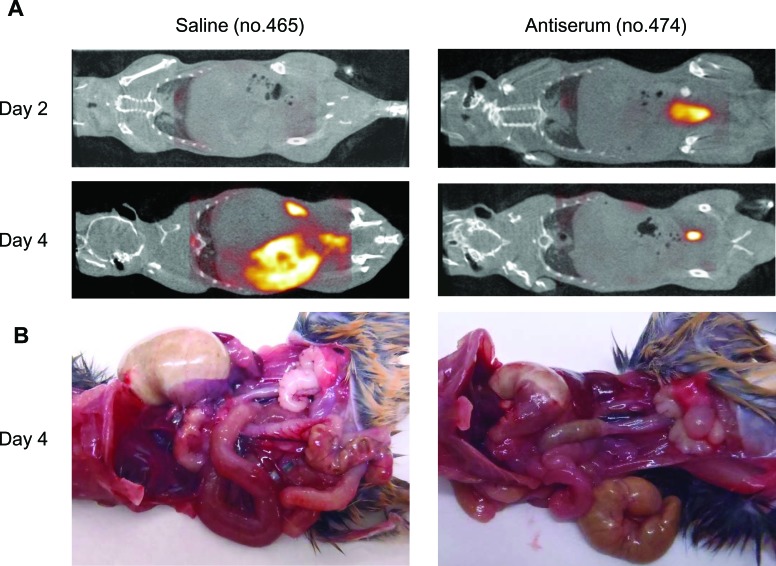
**A.** Representative whole body PET/CT images of SFTSV-infected A129 mice treated with saline (mouse no. 465) and antiserum (mouse no. 474) at 2 and 4 days pi. PET/CT images were acquired 30-60 min after intravenous injection of ^18^F-FDG (10 MBq in 400 μl of saline per mouse). **B.** Gross pathology of saline- and antiserum-treated A129 mice at 4 days pi. Mice were dissected after PET/CT imaging.

There was no ^18^F-FDG accumulation in the intestines of antiserum- and saline- treated mice at 2 day pi (Figure 3A, Video 3 and Video 4). The saline-treated mice exhibited ^18^F-FDG accumulation in the intestinal tract at 4 days pi (Figure 3A and Video 5). The gross pathology showed severe gastric and intestinal distension in addition to reduced cecum size (Figure 3B). On the other hand, the antiserum-treated mice showed no ^18^F-FDG accumulation in the intestinal tract (Figure 3A and Video 6), and there was no gastric or intestinal distension (Figure 3B). These observations suggest that ^18^F-FDG PET imaging can noninvasively monitor disease progression *in vivo* and can show the efficacy of therapeutics.

## DISCUSSION

This is the first report demonstrating the molecular imaging of infectious disease caused by SFTSV in a mouse model. Specific ^18^F-FDG uptake was observed in the intestinal tract of SFTSV-infected A129 mice.

Common symptoms of SFTS patients are nonspecific febrile illness and gastrointestinal tract disorders such as nausea, anorexia and diarrhea in the early phase of the disease, followed by progressive decline in platelets and white blood cells [1-3]. It was suggested that higher viral load and abnormally expressed cytokine profiles in the serum are associated with the disease severity of SFTS in human cases [14-16].

On the other hand, SFTSV-infected A129 mice did not have elevated body temperature (data not shown). We previously showed that SFTSV-infected A129 mice exhibited high levels of viral replication in tissue, such as the lung, spleen, liver, kidney, brain, and spinal cord [13]. However, SFTSV-infected A129 mice may not reflect abnormal cytokine expression following SFTSV infection, because expressed cytokines are closely associated with IFN-I response. Thus, it is controversial whether disease progression in a mouse model directly reflects the disease in human cases.

However, SFTSV-infected A129 mice showed apparent gastrointestinal disease. Mice showed acute weight reduction after 2 days pi [13] and the loss of appetite that led to decreased food and water intake probably caused it (data not shown). Thus, the clinical signs in mice may be relevant to the gastrointestinal symptoms in human cases during SFTSV infection. Pathological observations of SFTSV-infected A129 mice showed gastric and intestinal distensions, reduced stools in the intestine, erosion in the gastric mucosa. However, the pathological features in human SFTS cases have not been well described. Although, further information including the histopathological findings in human cases will be required to assess whether a mouse model actually reflects the gastrointestinal disease in human cases, we raise the possibility that PET imaging data will provide important clues to the *in vivo* studies of SFTSV infection and preclinical studies such as the assessment of drugs for SFTS treatment.

Interestingly, ^18^F-FDG accumulated in the intestinal tract but not in the stomach of SFTSV-infected mice. ^18^F-FDG is a glucose analogue that is absorbed by cells with a high-glucose demand. The FDG is moved by glucose transporters and is phosphorylated to ^18^F-FDG-6-phosphate once in cells, but it is not further metabolized in cells [17]. There have been several reports of ^18^F-FDG PET imaging for the detection of pathological conditions, such as tumors and inflammatory diseases, in the gastrointestinal tract [10, 18-20]. Rat models have shown that ^18^F-FDG uptake occurs in indomethacin-induced intestinal ulceration models, and ^18^F-FDG accumulation was found in inflammatory cells [21].

Viral infections and inflammatory reactions in the intestine may be correlated with specific ^18^F-FDG accumulation in SFTSV infected-mice. However, ^18^F-FDG accumulations were not observed in other sites of gastrointestinal tract and other organs. In particular, gross pathological changes were apparently present in the stomach, but ^18^F-FDG uptake was not observed. Although it is unclear why the specific ^18^F-FDG uptake was observed in the intestine but not in the stomach, the elucidation of the mechanism will provide interesting insights into the mechanism of gastrointestinal disease *in vivo* due to lethal infection with SFTSV.

Classical approaches to preclinical studies of pathogenic virus infections using animal models involve euthanasia and tissue dissection. As a result, it was impossible to follow the real-time dynamics of disease progression in the same individuals. Molecular imaging techniques are noninvasive and can provide insight into disease progression, prognosis, and responses to therapy in the same individuals. However, there are currently a limited number of reports using *in vivo* molecular imaging for highly pathogenic pathogens. Thus, molecular imaging is expected to play an increasing role in the pathological studies and the development of effective treatment strategies for infectious viruses including BSL-3 viruses.

## MATERIALS AND METHODS

### Virus and cells

The YG-1 strain of SFTSV was kindly provided by Ken Maeda at Yamaguchi University. The SFTSV stock virus was prepared from cell culture medium of Vero E6 cells. Vero E6 cells were maintained in Eagle's Minimal Essential Medium (EMEM; Nissui Pharmaceutical Co., Tokyo, Japan) containing 10% fetal bovine serum (FBS). The viral titers were determined using the focus forming assay described previously [13]. All experiments using live SFTSV were performed in a BSL-3 laboratory at Nagasaki University according to standard BSL-3 guidelines.

### Mice

A129 mice were purchased from B & K Universal Limited and were mated in the facility at Nagasaki University. Adult mice were subcutaneously inoculated with 10^6^ focus-forming units (ffu) of SFTSV diluted in EMEM containing 2% FBS. Animal experiments were performed in accordance with the recommendations in the Fundamental Guidelines for Proper Conduct of Animal Experiment and Related Activities in Academic Research Institutions under the jurisdiction of the Ministry of Education, Culture, Sports, Science and Technology. The Animal Care and Use Committee of the Nagasaki University approved all of the experimental protocols (approval number: 1210251024, 1401201115-6).

### Histopathological study

SFTSV-infected A129 mice were anesthetized and then perfused with 10% phosphate-buffered formalin at 3 days pi. The fixed tissues were routinely embedded in paraffin, sectioned, and stained with hematoxylin and eosin. Viral RNA was detected using ISH in formalin-fixed, paraffin-embedded (FFPE) sections using the AT-tailing method for high-sensitivity detection as described previously [22]. The AT-tailed oligonucleotide cocktail antisense probes L(5′-tcagggaagcaatgacagacgccttccatggtaatagggaatatatatatatatatatat-3′), M ctccttggatatgcaggcctcatgctactcaccaaatatatatatatatatatat-3′) and S(5′-cttctgtcttgctggctccgcgcatcttcacattgatagtatatatatatatatatatat-3′) segments of the viral RNA were used as probes. The reaction products were visualized in diaminobenzidine tetrahydrochloride and hydrogen peroxide, and the nuclei were lightly counterstained with hematoxylin.

### ^18^F-FDG-PET/CT imaging

PET/CT images were acquired using Triumph combined PET/SPECT/CT systems (TriFoil Imaging, Inc., CA, USA). SFTSV-infected A129 mice were administered approximately 10 MBq of ^18^F-FDG (Nihon Medi-Physics Co., Ltd. Kurume, Japan) intravenously *via* the tail vein. The mice were anesthetized with 1.5% isoflurane, and CT acquisitions were performed for anatomical reference. The PET acquisitions were performed for 30 min starting 30 min after ^18^F-FDG injection. The PET data were reconstructed using a 3D-Maximum-Likelihood Expectation Maximization (MLEM) algorithm (60 iterations). The acquired PET and CT data were processed using VIVID™ (TriFoil Imaging, Inc.) and OsiriX MD (FDA cleared, Pixmeo).

### Antiserum treatment in SFTSV-infected mice

Antiserum was collected from a patient who contracted SFTS in 2005. The serum collection was performed eight years after the patient recovered. The experiment using human serum was performed with the approval of the Ethics Committee of the Institute of Tropical Medicine, Nagasaki University (approval number: 140829129). The serum was diluted tenfold in saline, and 500 μl of diluent was intraperitoneally injected in mice 1, 24, 48 and 72 hours after inoculation with 10^6^ ffu of SFTSV. Saline was used for mock treatment. Our previous data indicated that antiserum treatment by 3 days pi completely protected mice against lethal infection. Thus, we observed PET imaging after the antiserum treatment to compare survivals between treated and non-treated mice. In addition, to examine the course of disease progression, we observed the sequential imaging in individual mice. Furthermore, to minimize the influence of the intravenous damages by the injection, we examined the mice every second day. As a result, we performed PET imaging at 2 and 4 days pi for antiserum treatment.

## SUPPLEMENTARY VIDEOS












